# Draft Genome Sequence of the Hydrocarbon-Degrading Bacterium *Alcanivorax dieselolei* KS-293 Isolated from Surface Seawater in the Eastern Mediterranean Sea

**DOI:** 10.1128/genomeA.01424-15

**Published:** 2015-12-10

**Authors:** Marta Barbato, Francesca Mapelli, Bessem Chouaia, Elena Crotti, Daniele Daffonchio, Sara Borin

**Affiliations:** a Department of Food, Environmental and Nutritional Sciences (DeFENS), University of Milan, Milan, Italy; b Biological and Environmental Sciences and Engineering Division (BESE). King Abdullah University of Science and Technology (KAUST), Thuwal, Saudi Arabia

## Abstract

We report here the draft genome sequence of *Alcanivorax dieselolei* KS-293, a hydrocarbonoclastic bacterium isolated from the Mediterranean Sea, by supplying diesel oil as the sole carbon source. This strain contains multiple putative genes associated with hydrocarbon degradation pathways and that are highly similar to those described in *A. dieselolei* type strain B5.

## GENOME ANNOUNCEMENT

Members of the *Alcanivorax* genus are important marine hydrocarbonoclastic bacteria detected in oil-impacted marine environments all over the world. Their number increases very quickly after oil spills, although it declines after only few weeks (see Head et al [1] and references therein). Representatives of this genus very efficiently use both branched and linear alkanes as energy sources for their growth, thanks to specific enzymes (mainly monooxygenases and hydroxylases) that catalyze the degradation of hydrocarbon molecules (1). *Alcanivorax dieselolei* was first isolated in 2001 from Bohai Sea surface waters in China and from deep-sea sediments of the Pacific Ocean (2). The *A. dieselolei* strain KS-293 presented in this study was isolated from surface waters of the Eastern Mediterranean Sea (34°42′E 34°00′N) by establishing enrichment cultures using the mineral marine medium ONR7a (3) and supplying diesel oil (1% [vol/vol]) as the sole carbon source.

The genomic DNA from *A. dieselolei* KS-293 was purified using the DNeasy blood and tissue kit (Qiagen, Milan, Italy).

Genome sequencing was carried out on an Illumina HiSeq 2000 platform by GATC Biotech (The Netherlands). The obtained 150 bp-long paired-end reads (~24 × 10^6^ reads, ~3 Gb) were assembled into the draft genome using Mira (version 4.1) (4). An automatic functional annotation of the predicted genes was performed using the RAST server (5) after using Glimmer as an open reading frame (ORF) caller. The assembly generated a draft genome of 4,790,658 bp (~500-fold coverage) and 57 contigs. The genome contained 4,445 predicted coding sequences, and the G+C content was 62.09%.

Despite the geographic distance of the isolation sites, the comparative analyses of the *A. dieselolei* KS-239 genome with the previously sequenced type strain *A. dieselolei* B5 (6) highlighted the presence of highly similar genes involved in the hydrocarbon degradation pathways. We detected three alkane monooxygenase-coding genes (AlkB) showing identities of 97%, 99%, and 99% with the AlkB genes belonging to *A. dieselolei* B5 (B5T_04393, B5T_00103, and B5T_00721, respectively). We also identified in *A. dieselolei* KS-293 two cytochrome P450-coding genes sharing identities of 100% and 97% with two of the three cytochrome P450 enzymes detected in the type strain B5 (B5T_02075 and B5T_02349, respectively). Moreover, two cyclohexanone monooxygenase and one flavin-binding family monooxygenase have been identified in the draft genome of strain KS-293, showing identities of 95%, 98%, and 97% with strain B5 AlmA genes (B5T_00657, B5T_02052, and B5T_00581, respectively). One haloalkane dehalogenase (DadB) with 97% identity with type strain B5 DadB (7) was also detected.

The *A. dieselolei* KS-293 genome was sequenced to provide a baseline for further transcriptomic analyses, with the aim of obtaining deep insights into the changes in hydrocarbon degradation metabolism occurring at the strain level under different environmental conditions (e.g., low-high hydrostatic pressure).

### Nucleotide sequence accession numbers.

The draft genome sequence of the *A. dieselolei* KS-293 strain was deposited at the European Nucleotide Archive (ENA) under the accession numbers CZHF01000001 to CZHF01000057.
